# Artificial intelligence in radiology and diagnostic imaging

**DOI:** 10.6026/973206300211891

**Published:** 2025-07-31

**Authors:** Devta Viswam, Ashish Abraham Francis, Sachin Dawale, Ranjani Kanakaraj, Yagvalkya Sharma, Lalit Karki, Shrinidhi Sivasubramaniam, Prashannalakshmi A.

**Affiliations:** 1 Department of Radiology, Caritas Hospital, Kottayam, India; 2 Institute of Internal Medicine, Madras Medical College, Chennai, India; 3 Department of Radiodiagnosis, Mahatma Gandhi Institute of Medical Science, Sevagram, Wardha, Maharashtra, India; 4 General Practitioner, Institute of Internal Medicine, Madras Medical College, Chennai, Tamil Nadu, India; 5 Department of Research, Kalp Research Work, Mathura, Uttar Pradesh, India; 6 Department of Radiology, Kathmandu Medical College, Kathmandu, Nepal India; 7 Institute of Internal Medicine, Madras Medical College, Chennai, India; 8 Department of Medicine, Tashkent Medical Academy, Urgench branch, Uzbekistan

**Keywords:** Artificial intelligence, machine learning, deep learning, radiomics, computer-aided detection, workflow optimization, diagnostic imaging

## Abstract

Artificial intelligence applications in radiology and diagnostic imaging encompass machine learning algorithms and deep neural networks that enhance image acquisition, analysis, and interpretation. Automated detection tools improve lesion conspicuity and quantification in modalities such as computed tomography, magnetic resonance imaging, and ultrasound, yielding greater diagnostic accuracy and consistency. AI-driven workflow optimization streamlines image reconstruction and prioritizes critical findings to accelerate clinical decision making and reduce reporting turnaround times. Integration of radiomics and predictive modeling facilitates noninvasive phenotyping of tissue characteristics and risk stratification for personalized patient management. Despite promising outcomes, challenges remain in algorithm generalizability, integration with picture archiving and communication systems, and regulatory approval pathways. Ethical considerations including data privacy, algorithmic bias, and explainability must be addressed to ensure safe and equitable deployment. This narrative review synthesizes current developments, evaluates clinical efficacy, and outlines future directions for AI in radiology and diagnostic imaging.

## Background:

Advances in radiology and diagnostic imaging have transformed disease detection and management by providing high resolution visualization of anatomical structures and physiological processes [1]. Traditional image interpretation relies on the expertise of radiologists to identify patterns indicative of pathology, yet interobserver variability and growing imaging volumes strain clinical workflows and can delay diagnosis [2]. Artificial intelligence offers the potential to enhance image quality through noise reduction and accelerated reconstruction algorithms while enabling automated lesion detection and quantitative feature extraction [3]. Early machine learning applications used handcrafted features to classify imaging findings but were limited by the need for manual feature selection and small training sets. Recent developments in deep learning, particularly convolutional neural networks, have demonstrated the ability to learn hierarchical imaging representations directly from raw data and to achieve performance on par with expert readers in tasks such as pulmonary nodule detection on computed tomography, breast lesion classification on mammography, and brain tumor segmentation on magnetic resonance imaging.

Integration of radiomics with clinical and genomic data further extends the role of diagnostic imaging from visual assessment to predictive modeling of treatment response and patient prognosis [4]. Despite these promising results, widespread clinical adoption is challenged by variations in imaging protocols, the need for large annotated datasets, regulatory considerations for software as a medical device, and concerns regarding algorithm transparency and data privacy [5]. This narrative review will explore the evolution of artificial intelligence in radiology, its current applications across imaging modalities, and the barriers that must be addressed to realize its full potential [6].

## Literature search strategy:

A comprehensive search of the literature was conducted to identify studies, reviews, and reports on artificial intelligence in radiology and diagnostic imaging published between January 2010 and June 2025 by querying PubMed, Embase, and IEEE Xplore using controlled vocabulary and free text terms such as artificial intelligence, machine learning, deep learning, radiomics, computer aided detection, image segmentation and diagnostic imaging [7]. After removal of duplicate records, titles and abstracts were screened to exclude case reports, conference abstracts lacking full data, non English publications, and studies without primary methodological or clinical outcomes [8]. Full texts of the remaining articles were then reviewed to include original research and comprehensive reviews that detailed algorithm development or validation, clinical performance studies, workflow integration evaluations, and implementation barriers [9]. Data were extracted on imaging modality, AI technique, dataset size and source, performance metrics such as sensitivity and specificity, clinical setting, and reported challenges. Reference lists of key papers were hand searched to capture additional seminal works [10]. Although this narrative review does not adhere to formal systematic review protocols, this transparent and reproducible search approach ensures a representative overview of current developments in AI for diagnostic imaging [11].

## Overview of artificial intelligence techniques:

Artificial intelligence in diagnostic imaging uses a range of computational methods that learn from data to perform tasks such as image classification, segmentation and clinical outcome prediction. Supervised learning approaches rely on labeled datasets in which expert annotations guide training of algorithms to recognize patterns associated with pathology [12]. Convolutional neural networks have become the standard for image classification because they automatically extract hierarchical features from raw pixel data [13]. In segmentation applications, fully convolutional architectures generate pixel level predictions to delineate structures such as organs or lesions with high precision [14]. Radiomics workflows compute large arrays of quantitative features such as texture, shape and intensity from regions of interest and then apply traditional machine learning classifiers to develop predictive models [15]. Transfer learning techniques use pretrained networks on large natural image repositories and then fine tune them on medical imaging data to overcome limited domain specific datasets [18]. Unsupervised learning methods including clustering and dimensionality reduction algorithms identify intrinsic patterns in imaging data without the need for labels and can reveal novel image phenotypes [16]. Reinforcement learning frameworks are under investigation to optimize image acquisition parameters and automate protocol selection in real time [17]. Recent advances in attention mechanisms and transformer architectures offer the potential to model long range dependencies within images and to integrate multiple data streams such as imaging and clinical records. Together, these artificial intelligence techniques provide a versatile toolkit for enhancing image interpretation and driving predictive analytics in radiology [18].

## Clinical applications and performance:

Artificial intelligence algorithms have been applied across a broad range of radiology tasks and have demonstrated performance that rivals or exceeds human experts in many settings. In chest imaging, convolutional neural networks trained on large collections of chest radiographs achieve high sensitivity and specificity for detection of pulmonary nodules and consolidation consistent with pneumonia [19]. In neuroimaging, deep learning models applied to magnetic resonance sequences accurately segment brain tumors and quantify lesion volumes with reproducibility that surpasses manual delineation. Mammography analysis with AI systems improves cancer detection rates while reducing false positive recalls by integrating lesion detection with risk stratification models [20]. Ultrasound applications include automated measurement of cardiac chamber dimensions and ejection fraction estimation from two dimensional cine loops, expediting echocardiographic reporting [21]. Performance metrics reported in clinical validation studies typically include area under the receiver operating characteristic curve values above 0.90 for classification tasks, Dice similarity coefficients above 0.85 for segmentation tasks, and reductions in reporting turnaround time of 30 percent or more when AI triage tools prioritize studies with critical findings [22]. Prospective trials have also begun to show improvements in diagnostic accuracy and workflow efficiency when AI assistance is integrated into radiology reading stations. These clinical applications illustrate both the versatility of artificial intelligence in diagnostic imaging and its potential to enhance patient care through improved accuracy consistency and efficiency [23].

## Workflow integration and implementation:

Adopting artificial intelligence in radiology requires seamless integration into existing clinical infrastructures including picture archiving and communication systems and reporting workstations. AI tools must support standard DICOM formats and integrate with vendor neutral archives to enable automated image routing and processing without adding manual steps [24]. User interfaces should present AI outputs such as heat maps segmentation overlays or quantitative measurements alongside original images to facilitate rapid expert review rather than replacing the radiologist. Automated notifications can be configured to flag studies with critical findings such as large pulmonary emboli or intracranial hemorrhage and prioritize their placement in reading queues [25]. Vendor supplied or homegrown governance frameworks govern model versioning performance monitoring and user feedback loops to ensure continuous improvement and safe deployment. Training programs for radiologists and technologists that cover AI fundamentals validation metrics and potential failure modes help build trust and encourage adoption [26]. Metrics for implementation success include changes in turnaround time's diagnostic accuracy and user satisfaction which are monitored through dashboards and periodic audits. Collaborative initiatives between IT department's clinical leadership and AI developers are critical to overcome regulatory hurdles data privacy requirements and to establish clear protocols for incident response when AI outputs conflict with human interpretation [27].

## Challenges and limitations:

Despite promising advances, implementation of artificial intelligence in radiology faces significant hurdles. Variability in image acquisition protocols across institutions can degrade model performance when algorithms trained on one dataset are deployed on another, highlighting the need for rigorous external validation and calibration. The scarcity of large curated and annotated datasets limits algorithm development and may introduce bias when training data lack representation of diverse patient populations and pathologies. Algorithm opacity and the black box nature of many deep learning models can undermine clinician trust and complicate error analysis when unexpected predictions occur. Integration issues involve the necessity for strong cybersecurity procedures to ensure patient data privacy and the demand for high-performance computing resources that are not necessarily present in all clinical environments. Regulatory channels for software as a medical device are changing but are still complex and jurisdiction-dependent, causing uncertainty among developers and healthcare providers. Lastly, medico-legal issues occur where AI results disagree with human interpretation, and there is a need for unambiguous rules of responsibility and accountability. Solutions to these issues will involve concerted efforts in data standardization, open model development, multidisciplinary cooperation, and the formulation of overall regulatory and ethical frameworks.

## Future directions and emerging trends:

The subsequent era of artificial intelligence in radiology will focus on explainable model development that sheds light on decision processes and ensures clinician trust. Federated learning methods that train models on disparate sources of data without exposure of raw images will facilitate generalization across institutions while maintaining patient confidentiality. Multimodal data integration, blending imaging with electronic health record data and genomic profiles, will deliver richer models for personalized diagnosis and prognosis. Optimizing real time image acquisition with reinforcement learning can decrease scan times and enhance patient comfort. Concurrently, low code and no code AI platforms will democratize tool creation so that radiology groups can customize algorithms for regional workflows. Lastly, cooperation among professional societies, the regulatory bodies and industry stakeholders will be critical to defining standardized evaluation criteria, data sharing agreements and ethical standards that facilitate safe and equitable use of AI in diagnostic imaging.

## Conclusion:

Artificial intelligence has evolved in a short space of time from research tools to clinical applications for improving the accuracy of image interpretation, lowering reporting times and facilitating personalized patient treatment. Addressing challenges in data heterogeneity, model explainability and interfacing with established infrastructure will be key to unlocking the full potential of AI in radiology. Ongoing multidisciplinary collaboration and adoption of strong governance frameworks will propel responsible innovation and ensure AI technologies benefit patients in a variety of healthcare environments.

## References

[1] Hosny A (2018). Nat Rev Cancer..

[2] Ahmed N (2021). Biomed Res Int..

[3] Le E.P.V (2019). Clin Radiol..

[4] Putra RH (2022). Dentomaxillofac Radiol..

[5] Wanjari M (2024). Neurosurg Rev..

[6] Heo M.S (2021). Dentomaxillofac Radiol..

[7] Hung K (2020). Dentomaxillofac Radiol..

[8] Föllmer B (2024). Nat Rev Cardiol..

[9] Schaffter T (2020). JAMA Netw Open..

[10] Kocak B (2023). Eur Radiol..

[11] Boeken T (2023). Diagn Interv Imaging..

[12] Yamada A (2023). Radiol Med..

[13] Cui Y (2022). Int J Environ Res Public Health..

[14] Mannil M (2020). Curr Cardiol Rep..

[15] Lecler A (2023). Diagn Interv Imaging..

[16] Yedavalli V.S (2021). Clin Imaging..

[17] Veziroglu E.M (2023). Semin Nucl Med..

[18] Ma P (2022). J Healthc Eng..

[19] Rohde S, Münnich N (2022). Orthopadie (Heidelb)..

[20] Moawad A.W (2022). J Comput Assist Tomogr..

[21] Sharma P (2020). J Thorac Imaging..

[22] Nakaura T (2023). Magn Reson Med Sci..

[23] Zhou B (2022). J Ultrasound Med..

[24] Barat M (2021). Jpn J Radiol..

[25] Hespel A.M (2022). Vet Radiol Ultrasound..

[26] Kocak B (2023). Eur Radiol..

[27] Hughes H (2023). Clin Radiol..

